# Percutaneous Approach to a Complicated Case of Nephrolithiasis in a Pregnant Woman: A Case Study

**DOI:** 10.1089/cren.2016.0040

**Published:** 2016-04-01

**Authors:** Giuseppe Giusti, Danilo Abate, Antonello De Lisa

**Affiliations:** Department of Urology, University of Cagliari, Cagliari, Italy.

## Abstract

***Background:*** Lithiasis during pregnancy can be a serious problem representing a danger to both the mother and the fetus. Surgical intervention is needed in approximately one-third of patients reporting pain despite analgesia and/or signs of persistent infection and obstruction, but there is a lack of consensus in the scientific literature as to the use of the most appropriate procedure to treat this condition.

***Case Presentation:*** We describe our experience in the treatment of a complicated reno-ureteral lithiasis in a 27-year-old patient in the first trimester of pregnancy. The patient had a calcified ureteral stent with associated stone formation in the right kidney and a bladder stone at the distal extremity of the stent. She was treated by a combined approach by percutaneous nephrolithotripsy and transurethral cystolithotripsy. The procedure we performed was effective.

***Conclusion:*** Our experience reinforces the feasibility and safety of the kidney stone removal by the percutaneous approach also in a pregnant patient and supports the recommendations of the European guidelines: “in experienced centers, where necessary, percutaneous nephrolithotripsy should be considered.” The technical precautions taken have proven to be valid and are supported by the current literature. Therefore, we feel they can be recommended.

## Introduction and Background

Kidney stones during pregnancy can be a serious problem as they pose a danger to the mother and the fetus. Renal colic, infection, and obstruction are associated with premature rupture of membranes, which carry an increased risk of morbidity and mortality to the newborn.^1^

The majority of pregnant patients with symptomatic calculi (50%–80%) pass their stones spontaneously.^2^ However, surgical intervention is needed in approximately one-third of patients, who report pain despite analgesia and/or show signs of persistent infection and obstruction.

There is a lack of consensus in the scientific literature as to the use of the most appropriate procedure to treat this condition. According to the European Association of Urology (EAU) Guidelines (2015), if spontaneous passage does not occur, or if complications develop, placement of a ureteral stent or a percutaneous nephrostomy tube is necessary. Unfortunately, these treatments are often poorly tolerated and require a number of replacements during pregnancy, given the high likelihood of rapid encrustation. To avoid these problems, according to the EAU guidelines, in the case of renal calculi or pushed-up upper ureteral calculi, retrograde endoscopic and percutaneous nephrolithotripsy may be performed during pregnancy.^3,4^ The decision rests with the individual surgeon and these procedures should be performed only in experienced centers. Therefore, according to the EAU guidelines, these procedures can be defined as feasible but cannot be recommended. We want to contribute to the scientific debate describing our experience in one case of complicated reno-ureteral lithiasis in a pregnant patient at the end of the first trimester.

## Presentation of Case

The patient is a 27-year-old woman, who had previously suffered from bilateral kidney stones, was hepatitis C virus (HCV) positive, and had a history of recreational and pharmacologic drug abuse.

In 2012 the woman had been first referred to our center with a complaint of right-side reno-ureteral colics associated with same-side hydronephrosis and fever (38°C). Abdominal CT scan showed an obstructive stone formation at the level of the ureteropelvic junction (13 mm) and an 11 mm stone in the middle calix.

We treated the patient with antibiotics and with the placement of a Double-J ureteral stent. The patient was discharged 2 days after the procedure. At the time of discharge, she was placed on waiting list for definitive treatment of the renal lithiasis.

However, all subsequent attempts to contact the patient by phone, starting from 1 month after discharge, were unsuccessful.

In October 2014, the patient returned to our center complaining of a recurrence of right-side reno-ureteral colics accompanied by some episodes of hematuria, irritation of the lower urinary tract, and high fever (as much as 39°C).

The patient was in the 13th week of pregnancy: renal and bladder echotomography revealed the presence of a stone formation of about 4 cm in the renal pelvis and in the mid and lower renal caliceal group that was encrusted on the calcified proximal end of the Double-J stent. The right-side kidney was also affected by second-degree hydronephrosis. Another large stone of about 4.5 cm was found at the distal (bladder) end of the stent, around its tail. There were also multiple calcifications along the ureteral tract.

The patient was admitted to our unit, and on the same day she underwent urgent pyelo-caliceal puncture of a posterior lower pole calix under ultrasound guidance, with positioning of a nephrostomy tube. On the third day after admission, fever had resolved, but the patient still reported flank and hypogastric pain and had marked symptoms of bladder irritation. The patient had also expressed concern about the need for frequent replacement of the nephrostomy tube.

To address these issues, we opted for a definitive solution, by means of percutaneous lithotripsy of the kidney and bladder stones and removal of the ureteral stent after the necessary lithotripsy of the stones along the ureter.

**FIG. 1.**
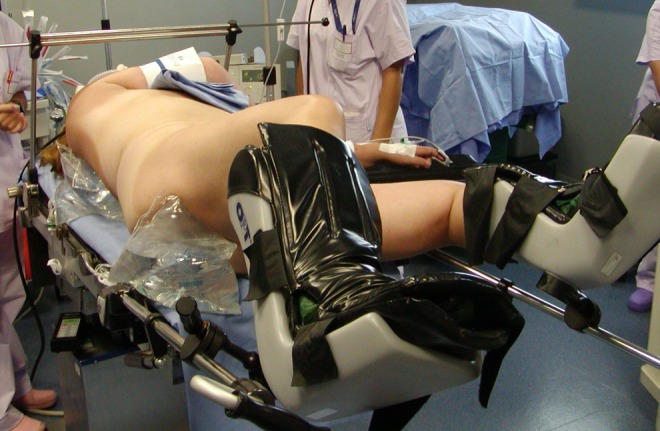
Position of the patient on the operating table: supine position slightly spread legs.

**FIG. 2.**
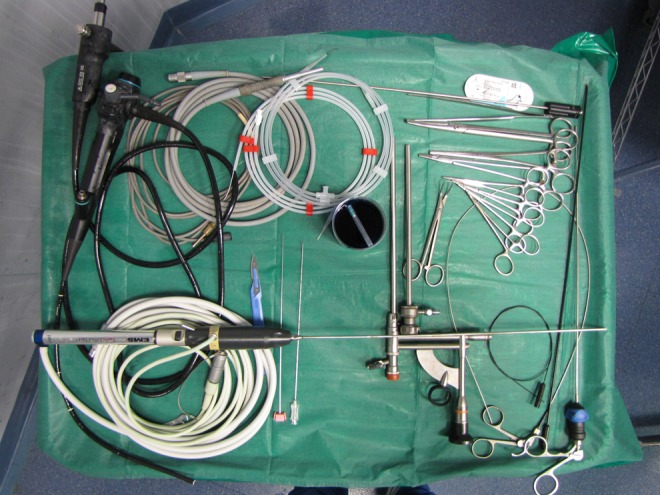
Surgical endoscopic cart for percutaneous nephrolithotomy procedures.

The patient was placed in a supine position with legs slightly spread (Fig. 1); we performed one-shot dilatation of the nephrostomy tract with Amplatz 22, inserted the operating cannula using ultrasound guidance, and then introduced a 20F rigid nephroscope (Fig. 2). A ballistic and ultrasonic energy probe was used to shatter the stone that was molded to the shape of the renal calix and pelvis and incorporated the proximal curled end of the Double-J ureteral stent. The lithotripsy lasted 50 minutes.

We then performed laser lithotripsy by means of antegrade flexible ureteroscopy on the many calcifications along the Double-J ureteral stent that anchored it to the ureter. This second phase lasted 20 minutes.

Maintaining the patient in the same position, the rigid cystoscope was then inserted into the bladder and the stone on the distal end of the Double-J stent was fragmented through ballistic and ultrasound energy probe. The ureteral catheter was then removed percutaneously. This third phase lasted 40 minutes.

Finally, a re-entry Malecot nephrostomy catheter 16F (Boston Scientific^©^) and a vesical catheter were put in place.

Total duration was 115 minutes. Five days after the procedure, the nephrostomy and vesical catheter were removed, and the following day the patient was discharged.

The patient completed her pregnancy with no further complications and delivered at term.

## Discussion and Literature Review

In the following lines, we would like to highlight some of the precautions we used. Organogenesis takes place during the first trimester of pregnancy, which is therefore the time when the embryo fetus is most sensitive to teratogens. To avoid exposing the fetus to ionizing radiations, we used ultrasound for diagnostic purposes and during the surgical procedure. As suggested by the EAU's 2015 guidelines, we opted for a conservative first-line treatment. The patient already had a calcified ureteral stent *in situ*, therefore, the only feasible choice, in this case, was a pyelo-caliceal puncture and the placement of a nephrostomy tube. Since the patient was pregnant and lithotripsy had to be performed on the kidney and bladder stones, we opted for the supine position with slightly spread legs to ensure access to the urethra. This position allows easy percutaneous lumbar and transurethral access and reduces certain anesthesiology problems associated with the prone position, which are typically heightened in pregnant and obese patients.

**FIG. 3.**
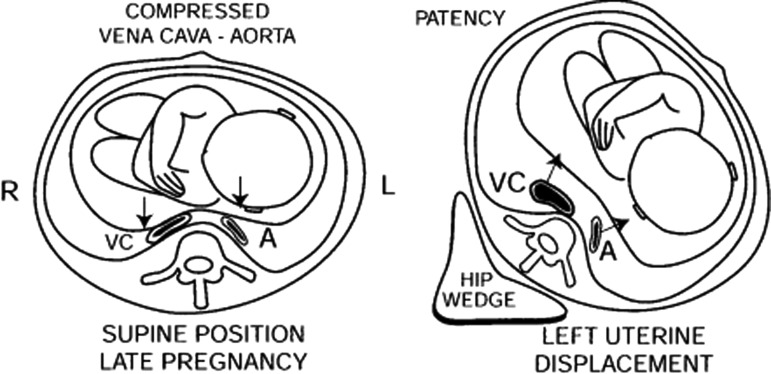
Compression of the aorta (A) and vena cava (VC) when the pregnant patient is in the supine position. Both vessels are instead patent when the patient lies on the left side. *Source:* Drips et al.^5^

However, the pure supine position causes in pregnant patients compression by the uterus of both the vena cava and the aorta. This complication is more frequent in later pregnancy, when the uterus is larger. As in this case, the kidney to be treated was the right-side one, the patient was placed slightly turned on the left side with the pregnant uterus tilted to the left. Thanks to this choice, the known problem of aorta and vena cava compression did not occur (Fig. 3).

To date, the literature has only four published reports (Kavoussi; Shah; Tóth; and Fregonesi) of percutaneous nephrolithotripsy in pregnant patients. Our case report confirms that the approach we used is feasible and safe and supports the recommendations of the European guidelines: in experienced centers, where necessary, more invasive approaches like percutaneous nephrolithotripsy should be considered in the treatment of renal lithiasis during pregnancy. The technical precautions taken have proven to be valid; therefore, we believe they can be recommended in clinical practice.

## Abbreviations Used

CT: computed tomography
EAU: European Association of Urology
